# Maternal and Neonatal Outcomes Associated with Mild COVID-19 Infection in an Obstetric Cohort in Brazil

**DOI:** 10.4269/ajtmh.22-0421

**Published:** 2022-10-17

**Authors:** Carolina A. D. Santos, Gentil G. Fonseca Filho, Manoella M. Alves, Erianna Y. L. Macedo, Monise G. de A. Pontes, Artemis P. Paula, Carolina T. R. Barreto, Felipe N. Zeneide, Andréia F. Nery, Reginaldo A. O. Freitas, Lília D’Souza-Li

**Affiliations:** ^1^ University of Campinas, UNICAMP, Faculty of Medical Science, Campinas, São Paulo, Brazil;; ^2^ Anita Garibaldi Center for Education and Research in Health, Santos Dumont Institute, Macaíba, Rio Grande do Norte, Brazil;; ^3^ Federal University of Rio Grande do Norte, Santa Cruz, Rio Grande do Norte, Brazil;; ^4^ Federal University of Rio Grande do Norte, Natal, Rio Grande do Norte, Brazil;; ^5^ University of Campinas, UNICAMP, Faculty of Medical Science, Department of Pediatrics, Campinas, São Paulo, Brazil

## Abstract

Previous coronavirus epidemics were associated with increased maternal morbidity, mortality, and adverse obstetric outcomes. Reports for SARS-CoV-2 indicate that the obstetric population is at increased risk for severe illness, although there are still limited data on mild COVID-19 infection during pregnancy. To determine the association between mild COVID-19 infection during pregnancy, and maternal and neonatal outcomes, we performed a prospective cohort study among pregnant women with COVID-19 and a control group. Postnatal depressive symptoms were assessed using the Edinburgh Postnatal Depression Scale. We recruited 84 pregnant women with mild COVID-19 and 88 pregnant women without COVID-19. All participants were unvaccinated. The most common acute COVID-19 symptoms were headache (82.1%), loss of smell (81%), and asthenia (77.4%). The median duration of long COVID symptoms was 60 days (interquartile range, 130). Pregnant women with a COVID-19 diagnosis were at greater risk for obstetric ultrasound abnormalities—mainly, fetal growth restriction (relative risk [RR], 12.40; 95% CI, 1.66–92.5), premature birth (RR, 2.62; 95% CI, 1.07–6.43), and postpartum depression (RR, 2.28; 95% CI, 1.24–4.21). Our results alert clinicians to the consequences of COVID-19 during pregnancy, even in mild cases, given the increased risk of ultrasound abnormalities, premature birth, long COVID symptoms, and postpartum depression. National guidelines on preventive measures and treatments should be based on scientific evidence, including attention to the impact on health and family needs during and after the COVID-19 pandemic.

## INTRODUCTION

COVID-19, an infectious disease caused by SARS-CoV-2, was declared a global pandemic by the WHO in March 2020.^1^ COVID-19 has infected more than 500 million people and killed more than 6 million worldwide,^2^ causing disruptions to essential health services, food security, education, and employment. Previous coronavirus epidemics were associated with increased maternal morbidity, mortality, and adverse obstetric outcomes.^3^ Reports have shown that the obstetric population is in fact at increased risk for severe illness associated with COVID-19,^4^^,^^5^ although data are still limited on COVID-19 in this population.^6^^–^^8^

Since the beginning of the pandemic, Brazilian scientists have tried to bring attention to the high mortality rate among pregnant women with COVID-19.^8^^,^^9^ As of April 2022, at least 22,048 pregnant or postpartum women confirmed an infection for SARS-Cov-2, and 2,026 (9.2%) died of COVID-19, with 20% of the patients having not even been admitted to an intensive care unit.^10^ The extent of the impact of COVID-19 on maternal and perinatal outcomes in obstetric patients in Brazil remains largely unknown, especially in populations living with persistent inequalities of income, age, race, and geographic location.

In a living systematic review and meta-analysis in pregnant women, compared with women without COVID-19, women with COVID-19 had a greater risk of maternal death, admission to the intensive care unit, and preterm birth. Neonates had a greater risk of admission to a neonatal intensive care unit than neonates born to women without the disease.^11^ Although uncommon, some reports showed the possibility of vertical transmission.^12^^–^^14^ Also, some studies have shown the negative mental, economic, and social impacts the pandemic has brought to the obstetric population.^15^^–^^17^ Understanding COVID-19 infection in pregnancy and newborns is essential to defining research priorities, surveillance programs, and future public policies.

We performed a prospective cohort study among pregnant women with COVID-19 and a control group in northeastern Brazil to determine the association between COVID-19 and pregnancy and neonatal outcomes, long COVID symptoms, and maternal depression.

## MATERIALS AND METHODS

### Research setting.

This was a prospective cohort carried out at a referral center for pregnant women with diseases susceptible to vertical transmission in northeastern Brazil. Study participants initially included 88 pregnant women with laboratory confirmation of COVID-19 infection between March 18, 2020 and July 31, 2021. Subsequently we excluded four patients: one with moderate illness and three with severe illness, leaving 84 participants with mild COVID-19. After providing informed consent, the participants answered a questionnaire about epidemiological data, presence and duration of clinical symptoms, type of treatment received, need for hospitalization, oxygen support, and intensive care. During prenatal follow-up at the clinic, patients underwent clinical consultations with a multidisciplinary team, ultrasound, and laboratory tests recommended by the national guidelines for antenatal visits. On the day of their first appointment at the referral center, each pregnant woman was paired with a patient in the control group (*n* = 88) of the same age. All control subjects received prenatal care at the same clinic and had no history of suspected or confirmed COVID-19. All data (clinical, laboratory, ultrasound) from both groups and newborns were collected prospectively from medical records. During follow-up, patients who reported persistent symptoms during the post-COVID-19 period—3 weeks in mild to moderate cases, or after discharge in patients with severe or critical illness if the patients were still in acute care after 3 weeks^18^—were monitored in subsequent consultations and during the postpartum period about the duration of symptoms. Symptoms that may be related to pregnancy (e.g., shortness of breath) were only considered if they persisted during the postpartum period. Postnatal depressive symptoms were assessed at the first postpartum consultation using the Edinburgh Postnatal Depression Scale (EPDS),^19^ and the cutoff point for screening for postpartum depression was ≥ 11. Patients from both groups had at least six prenatal and two postpartum visits at the clinic (more visits if it was necessary). On average, patients who had COVID-19 were monitored for 6 months after the infection (longer if they persisted with long COVID symptoms). The following variables were recorded for both groups: maternal age, prior medical comorbidities, first- or second-trimester miscarriage, fetal death or neonatal death, preterm birth (< 37 weeks), ultrasound abnormalities, mode of delivery, Apgar at 1 and 5 minutes, and infant birthweight, length, and head circumference. For fetal growth assessment via ultrasound, the International Society of Ultrasound in Obstetrics and Gynecology guidelines were used.^20^ Small-for-gestational-age was considered when the estimated fetal weight or abdominal circumference was less than the 10th percentile, and late fetal growth restriction (FGR) when the estimated fetal weight or abdominal circumference was less than the third percentile or when at least two of the three following criteria were met: abdominal circumference/estimated fetal weight less than the 10th percentile, a decrease in fetal growth velocity (abdominal circumference/estimated fetal weight) greater than two quartiles, or alterations on Doppler velocimetry (uteroplacental and fetoplacental). Oligohydramnios was defined as a maximum vertical pocket shorter than 2 cm.^21^ The Strengthening the Reporting of Observational Studies in Epidemiology guideline and checklist were followed.^22^

### Statistical analysis.

All statistical analyses were performed using SPSS Statistics software (version 26; IBM Corp., Armonk, NY). Categorical variable values were reported as numbers with the relative percentage, and continuous variables were expressed using mean SDs if they were normally distributed, and median and interquartile ranges if they were not. The χ^2^ test or Fisher’s exact test was used to estimate the association between categorical variables, and Student’s *t*-test for independent samples or the Mann–Whitney *U* test was used for continuous variables as appropriate. Statistical significance was considered at *P* < 0.05.

## RESULTS

**Figure 1.**
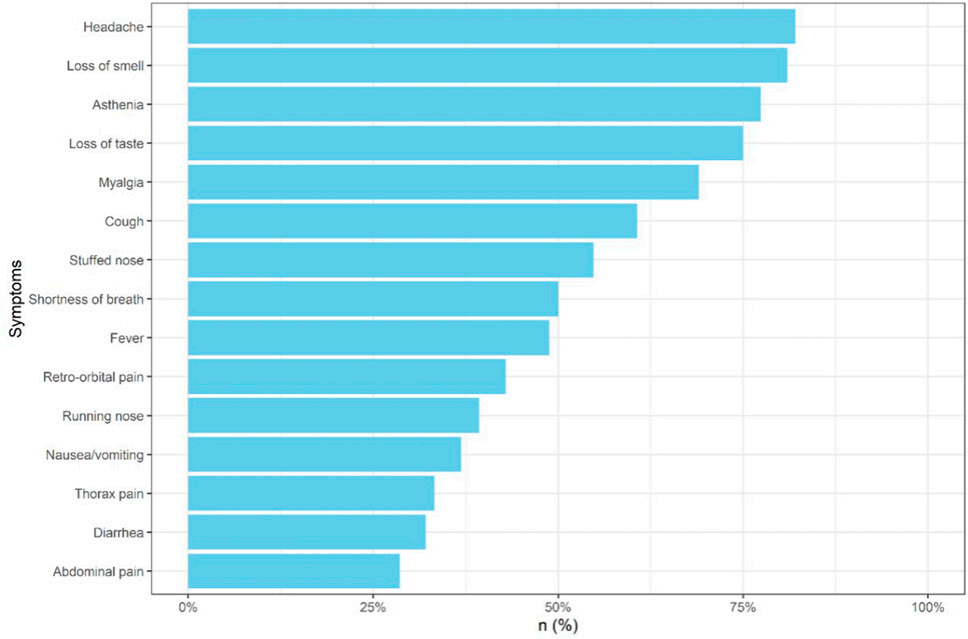
Frequency of acute symptoms reported by Brazilian pregnant women with confirmed mild COVID-19 infection. This figure appears in color at www.ajtmh.org.

All the participants with confirmed COVID-19 (*N* = 84) had at least one symptom before a reverse transcription–polymerase chain reaction or serological test was performed (Figure 1), were unvaccinated at the time of the infection, and were classified as mild illness according to the NIH disease severity classification.^23^ The most common reported symptoms were headache (82.1%), loss of smell (81%), and asthenia (77.4%). Fever occurred in 41 patients (48.8%), and cough in 51 (60.7%). Participants were between 5 and 40 weeks pregnant when infected. Twenty patients (23.8%) had the infection in the first trimester, 43 (51.2%) in the second, and 21 (12.2%) in the third. Twenty-four patients (28.6%) patients used a “COVID kit” prescription.^24^ The COVID kit included an off-label prescription of two or more of the following medications for outpatients in the first days of symptoms: chloroquine, hydroxychloroquine, azithromycin, dexamethasone (or other systemic glucocorticoids), nitazoxanide, ivermectin, and anticoagulants.

Maternal characteristics and obstetric/perinatal outcomes between the COVID-19 patient group and the control group are reported in Table 1. The comorbidities in both groups included chronic hypertension, gestational hypertension, pregestational diabetes, gestational diabetes, thyroid disease, obesity, and HIV. No miscarriages, fetal deaths, or neonatal deaths occurred in either group. Of the patients with completed pregnancies, 15 of 80 (18.8%) gave birth prematurely in the patient group compared with 6 of 84 (7.1%) in the control group (*P* = 0.026; relative risk [RR], 2.62; 95% CI, 1.07–6.43). Rates of cesarean delivery, Apgar scores at 1 and 5 minutes, average birth weight, length at birth, and head circumference were similar in both groups. All preterm births were classified as moderate to late, and within the COVID-19 group, 66.7% (10 of 15) of the infections occurred in the second trimester, 26.7% (4 of 15) in the first, and 6.7% (1 of 15) in the third. Only one patient went into labor during acute SARS-CoV-2 infection.

**Table 1:** Clinical characteristics, and maternal and perinatal outcomes among women with and without a COVID-19 diagnosis

Characteristic and outcome	COVID-19 group	Control group	*P *value*
Maternal age, years; median (IQR)	29.85 (10)	29.53 (9)	0.751
Parity, *n*/*N* (%)			
Nulliparous	35/84 (41.7)	22/88 (25)	0.013
Primiparous	21/84 (25)	31/88 (35.2)	0.144
Multiparous	28/84 (41.7)	35/88 (39.8)	0.844
Comorbidities, *n*/*N* (%)	34/84 (40.5)	35/88 (39.8)	0.920
Obstetric ultrasound abnormalities,† *n*/*N* (%)	11/47 (23.4)	1/53 (1.9)	< 0.001
Cesarean delivery, *n*/*N* (%)	52/80 (65)	50/84 (59.5)	0.470
Preterm birth (< 37 weeks), *n*/*N* (%)	15/80 (18.8)	6/84 (7.1)	0.026
Small for gestational age,‡ *n*/*N* (%)	7/80 (8.8)	2/84 (2.4)	0.073
Apgar score at 5 min < 7, *n*/*N* (%)	6/77 (7.8)	4/84 (4.8)	0.426
Postpartum depression,§ *n*/*N* (%)	18/40 (45)	12/61 (19.7)	0.006
Gestational age at birth, weeks; median (IQR)	38.2 (2.1)	38.7 (1.9)	0.054
Birthweight, g; mean (SD)	3,176.4 (519.5)	3,266.5 (461.2)	0.502

IQR = interquartile range.

* The χ^2^ test or Fisher’s exact test was used for categorical variables; Student’s *t*-test or the Mann–Whitney *U* test was used for continuous variables.

† Patients without comorbidities in both groups and at least 2 ultrasounds performed by the same professional.

‡ Less than the 10th percentile (International INTERGROWTH-21st Newborn Size Standards).

§ Edinburgh Postnatal Depression Scale score ≥ 11.

For the ultrasound analysis, we selected only those patients who had no comorbidities and who underwent at least two ultrasounds with the same professional in our service (47 in the patient group versus 53 in the control group). The presence of ultrasound abnormality was more frequent in pregnant women who had COVID-19 (11 of 47, 23.4%) compared with the control group (1 of 53, 1.9%; *P *≤ 0.001; RR, 12.40; 95% CI, 1.66–94.49) (Table 2). Four participants in the patient group presented late FGR, two patients had the infection in the second trimester and two in the third trimester. One patient in the patient group had abnormal fetal morphology (ventriculomegaly) 5 weeks after her COVID-19 infection. Reverse transcription–polymerase chain reaction of the amniotic fluid was negative for SARS-CoV-2, and karyotype results were normal.

**Table 2:** Obstetric ultrasound abnormalities among low-risk pregnancies

Abnormality	COVID-19 group (*n* = 47), *n *(%)	Control group (*n* = 53), *n *(%)
Fetal growth*		
Fetal growth restriction	4 (8.5)	1 (1.9)
Small for gestation age	3 (6.4)	0 (0)
Amniotic fluid, oligohydramnios†	3 (6.4)	0 (0)
Morphology	1 (2.1%)	0
Total‡	11 (23.4%)	1 (1.9%)

* International Society of Ultrasound in Obstetrics and Gynecology Guidelines for ultrasound assessment of fetal biometry and growth.

† Single deepest pocket < 2 cm.

‡ Fisher’s exact test, *P* < 0.001.

**Figure 2.**
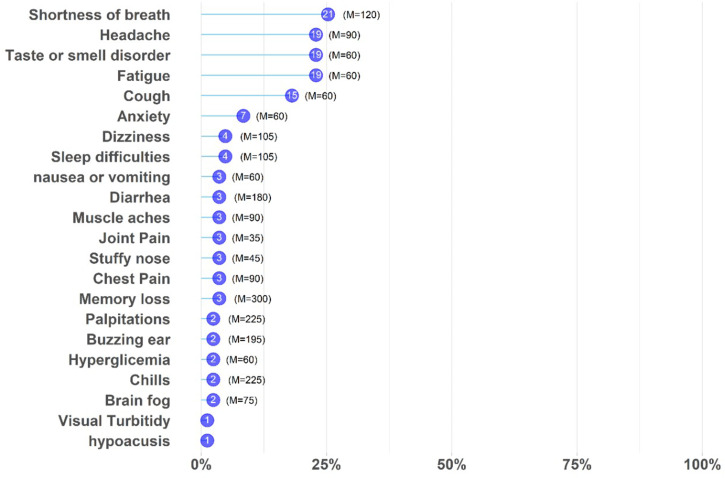
Frequency of long COVID symptoms among Brazilian pregnant women. Symptoms (*y*-axis) vs. frequency (*x*-axis) of pregnant women with long COVID. Purple circles show the number of women with symptoms. M= median number of days for each symptom. This figure appears in color at www.ajtmh.org.

Eighty-three participants in the patient group attended the follow-up for post-COVID-19 symptoms, and 63 of them (75.9%) presented long COVID, with the most common symptoms being shortness of breath (*n *= 21, 25.3%), fatigue (*n *= 19, 22.9%), headache (*n* = 19, 22.9%), and loss of taste and/or smell (*n *= 19, 22.9%) (Figure 2). The median duration of long COVID was 60 days (interquartile range, 130). Patients who took glucocorticoids from the COVID kit presented an increased risk of fatigue (*P* = 0.006; RR, 6.92; 95% CI, 1.70–28.07).

Forty patients in the patient group and 61 in the control group responded to the EPDS. In the patient group, 18 women (45%) exceeded the cutoff for postnatal depression compared with 12 (19.7%) in the control group (*P* = 0.006; RR, 2.28; 95% CI, 1.24–4.21).

## DISCUSSION

In this prospective COVID-19 obstetric cohort, we identified that pregnant women, mildly symptomatic, presented a greater risk for long-term complications, including ultrasound abnormalities, preterm birth, and postnatal depression. To our knowledge, no observational studies have been published to understand the impact of mild COVID-19 cases during pregnancy in Brazil.

The identified risk of having an ultrasound abnormality among COVID-19 patients without comorbidities compared with the control group could be explained by the effect of SARS-CoV-2 on the physiology of placental function. The development of FGR has been described with other SARS infections^3^ and other virus infections during pregnancy.^25^ A systematic review of placental histopathology after SARS-CoV-2 infection reported a high incidence of placental lesions, suggesting hypoperfusion and inflammation.^26^ Di Mascio et al.^3^ reported that of 41 pregnant women hospitalized with severe COVID-19, a 11.7% FGR rate was noted. However, it is not clear what criteria were used for FGR and if they also included small-for-gestational-age in the rate. We used the most restrictive criteria, and the difference in the disease severity reported by Di Mascio et al.^3^ and our cohort makes it difficult to compare their findings to our results. Oligohydramnios has been associated with various pathogenic infections during pregnancy,^27^ and we found a prevalence of 6% in the patient group—well more than the 0.2% and 1.5% described in low- to middle-income countries, respectively, for the general population.^28^ In a cohort of more than 5,000 pregnant women, perinatal SARS-CoV-2 transmission was estimated at 2.7%, mainly among infants born to women with infection close to the delivery.^29^ In our cohort we had one fetus diagnosed with moderate ventriculomegaly during the second trimester—5 weeks after a mild COVID-19 infection—and we excluded other potential causes (toxoplasmosis, rubella, cytomegalovirus, herpes simplex virus [TORCH], parvovirus B19, syphilis, and Zika virus infection). Ventriculomegaly was not described in SARS-CoV-2 infection; however, it has been reported with other viruses that have the potential for vertical transmission.^30^

An analysis of approximately 400,000 women age 15 to 44 years with symptomatic COVID-19 in the United States described cough and headache as the most frequent signs and symptoms.^4^ In line with other studies, headache was the most prevalent symptom in ours. Loss of smell, however, was found in 81% of our participants, whereas other studies reported only 21.5%,^4^ 24% among mild cases^31^ in another, and not reported^32^ in yet another. These discrepancies may be a result of different SARS-CoV-2 variants or differences in the genetic background of the population, as phenotypic expression of bitter taste receptors appeared to be associated with symptoms, immune response, clinical course, and recovery from COVID-19 infection.^33^

Several studies in different countries have shown the long-term impact of COVID-19 on an individual’s health.^34^ Some research reported persistent symptoms extending beyond the period of initial illness or hospitalization.^18^ In the United Kingdom, an estimated 20% of all people who tested positive for COVID-19 exhibited symptoms for 5 weeks or longer, and 10% exhibited symptoms for 12 weeks or longer.^34^ The prevalence of long COVID symptoms among pregnant women in our study was almost 80%, with more than one third presenting persistent symptoms postpartum. These findings are a warning of the impact of long COVID on this population and highlight the importance of providing adequate support for patients in need of long-term care. No other study has yet discussed the rates of long COVID in pregnancy or postpartum populations, rendering us unable to compare these results with others published. The relationship between early corticosteroid prescriptions and post-acute symptoms has not yet been determined, but corticosteroid treatment in the first days of SARS-CoV-2 infection delayed viral clearance significantly.^35^

It is worrisome that patients with mild symptoms received a prescription of “COVID kit treatments” that included medications not recommended during pregnancy. In a population-based Canadian pregnancy cohort,^36^ a study evaluating the safety of medication used, patients exposed to dexamethasone had an increased risk of prematurity, and the use of dexamethasone or azithromycin during the first trimester was associated with congenital malformations. Pregnant populations have been immensely affected during this pandemic,^37^ and previous respiratory virus epidemics,^38^ with our data exemplifying the chaotic national response, with nearly one third of the participants receiving prescriptions for unproven drugs against COVID-19. After 2.5 years of the pandemic, Brazil still faces multiple challenges, including the use of drugs that show no significant benefits in preventing or treating COVID-19, despite advice from infectious diseases societies.^39^ Educational interventions aimed at improving peoples’ ability to make informed health choices are needed desperately.^24^ Similarly, national guidelines on preventive measures and treatments should be based on scientific evidence, including attention to the impact on health and family needs during and after the COVID-19 pandemic. Public health institutions should prioritize pregnant women in COVID-19 vaccine programs, and the professionals who care for them should recommend and advocate for the COVID-19 vaccine to reduce vaccine hesitancy.^40^

In our study, pregnant patients with mild COVID-19 were at a greater risk of preterm birth and prematurity. This risk was not associated significantly with any comorbidity, the use of a COVID kit, parity, or maternal depression. Other studies have already established the association between COVID-19 in pregnancy and premature birth, but they were mainly hospital-based cases with severe infection during the third trimester of gestation, underrepresenting patients with earlier infection and mild symptoms.^7^^,^^29^^,^^41^

Despite not yet knowing the full impact on maternal mental health during the pandemic, maternal depression leads to a negative impact on the bond between mother and baby,^42^ and is linked to a greater risk of health problems, developmental delays, and behavioral problems in children.^43^ The EPDS scores from both the patient and control groups showed greater prevalence of postpartum depression than in pre-pandemic studies for the northeast region in Brazil.^44^ Similarly, a study evaluating postpartum depression among new mothers during the pandemic in England and Canada using the EPDS found notably greater levels of depressive symptoms among the group compared with pre-pandemic prevalence.^15^^–^^17^ Our study data corroborated these findings, and also showed that, in the group of pregnant women who had COVID, the incidence of depression was even greater. The high prevalence of maternal depression in our study emphasizes the need to protect maternal mental health, both during the ongoing COVID-19 pandemic and beyond.^15^ The research NEUROCOVID, of patients with mild respiratory symptoms, found that anxiety and cognitive impairment were manifested by 28% to 56% of COVID-19 convalescent individuals, and were associated with orbitofrontal cortical atrophy, which could contribute to the high prevalence of depression among recovered COVID-19 patients.^45^

Our study has some limitations. It was conducted in a single health-care center, and the number of participants in both groups was small, limiting the ability to identify differences between subgroups. The participants in our study were a convenience sample of pregnant women with COVID-19, as spontaneous demand or referred to the center, which could leave more vulnerable groups underrepresented. Because the participants were recruited from 2020 to 2021, they were unvaccinated and mainly infected with the variants B.1.1.28, B.1.1.33, P1, and P2.^46^ Outcomes may vary from other variants or vaccination status.

Our findings indicate that mild COVID-19 infection can have long-term consequences for maternal and child health. Future research should consider these findings when investigating how the infection is affecting the health of these women and their children, and the possible risks of SARS-CoV-2 infection for patients’ offspring.
